# Modeling and empirical validation of long‐term carbon sequestration in forests (France, 1850–2015)

**DOI:** 10.1111/gcb.15004

**Published:** 2020-02-13

**Authors:** Julia Le Noë, Sarah Matej, Andreas Magerl, Manan Bhan, Karl‐Heinz Erb, Simone Gingrich

**Affiliations:** ^1^ Institute of Social Ecology (SEC) Department of Economics and Social Sciences University of Natural Resources and Life Sciences Wien Austria

**Keywords:** carbon, climate change, forest, forest transition, France, land‐use, long‐term, modeling

## Abstract

The development of appropriate tools to quantify long‐term carbon (C) budgets following forest transitions, that is, shifts from deforestation to afforestation, and to identify their drivers are key issues for forging sustainable land‐based climate‐change mitigation strategies. Here, we develop a new modeling approach, CRAFT (CaRbon Accumulation in ForesTs) based on widely available input data to study the C dynamics in French forests at the regional scale from 1850 to 2015. The model is composed of two interconnected modules which integrate biomass stocks and flows (Module 1) with litter and soil organic C (Module 2) and build upon previously established coupled climate‐vegetation models. Our model allows to develop a comprehensive understanding of forest C dynamics by systematically depicting the integrated impact of environmental changes and land use. Model outputs were compared to empirical data of C stocks in forest biomass and soils, available for recent decades from inventories, and to a long‐term simulation using a bookkeeping model. The CRAFT model reliably simulates the C dynamics during France's forest transition and reproduces C‐fluxes and stocks reported in the forest and soil inventories, in contrast to a widely used bookkeeping model which strictly only depicts C‐fluxes due to wood extraction. Model results show that like in several other industrialized countries, a sharp increase in forest biomass and SOC stocks resulted from forest area expansion and, especially after 1960, from tree growth resulting in vegetation thickening (on average 7.8 Mt C/year over the whole period). The difference between the bookkeeping model, 0.3 Mt C/year in 1850 and 21 Mt C/year in 2015, can be attributed to environmental and land management changes. The CRAFT model opens new grounds for better quantifying long‐term forest C dynamics and investigating the relative effects of land use, land management, and environmental change.

## Graphical abstract

The CRAFT (CaRbon Accumulation in ForesT) model was developed to assess the carbon dynamics in French forests at the regional and national scale from 1850 to 2015. Model outputs were compared to empirical data of C stocks in forest biomass and soils, available for recent decades from inventories, and to a long‐term simulation using a bookkeeping model. The CRAFT model reliably reproduces C‐fluxes reported in inventories, in contrast to a widely used bookkeeping model which strictly only depicts C‐fluxes due to wood extraction. The difference between the bookkeeping and CRAFT model can be attributed to environmental and land management changes.

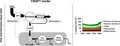

## 1 INTRODUCTION

Forests play a pivotal role in the global carbon (C) cycle and in mitigating climate change through C sequestration in biomass and soils (Bastin et al., 2019; Bonan, 2008; Canadell & Raupach, 2008; Erb et al., 2018; IPCC, 2019; Pan et al., 2011). They have been identified as the main terrestrial C sink, absorbing the equivalent of c. 18% of the C emissions from fossil fuel combustion. Forests not only contribute to climate change mitigation, they also provide, among other services, wood for fuel, timber, and paper industries. Although human land use, such as agriculture, is competing with forests (Curtis, Slay, Harris, Tyukavina, & Hansen, 2018; DeFries, Rudel, Uriarte, & Hansen, 2010; Pendrill, Persson, Godar, & Kastner, 2019), the deforestation rate has been declining at the global scale since the 2000s and some deforesting countries have even started afforesting (Keenan et al., 2015; Meyfroidt & Lambin, 2011). Forest transitions, that is, shifts from net deforestation to net reforestation at the national scale, have occurred in many early industrialized countries since the early 20th or even 19th century (Meyfroidt & Lambin, 2011), while more recently, forest transitions can also be observed in some tropical countries of the global South (Keenan et al., 2015; Southworth, Nagendra, & Cassidy, 2012). In Europe and North America, such forest transitions have led to increasing C sequestration in forest soils and biomass (Birdsey, Pregitzer, & Lucier, 2006; Ciais et al., 2008; Nabuurs, Schelhaas, Mohren, Frits, & Field, 2003).

While initially, the forest transition literature focused mainly on forest area (Mather, 1992), recent work has conceptualized forest transitions in terms of both forest area and forest C density (Houghton et al., 2000; Kauppi et al., 2006; Köhl et al., 2015). Growing forest area and forest C stocks may result from a variety of drivers. Reduced wood extraction (enabled, e.g., by forest protection and/or wood fuel substitution) can result in vegetation thickening and increased C stocks in forests (Gingrich et al., 2019; Kauppi et al., 2006; Magerl, Le Noë, Erb, Bhan, & Gingrich, 2019). Changes in growth conditions owing to environmental changes such as climate change, and changes in forest secondary uses may have similar effects (Erb et al., 2013). Forest area expansion is often enabled by agricultural intensification and/or increasing biomass imports (Meyfroidt & Lambin, 2009). As forest recovery can significantly contribute to climate change mitigation, a better understanding of forest recovery processes is required to tap the climate‐change mitigation potentials of reforestation (Erb, Gingrich, Krausmann, & Haberl, 2008; Erb et al., 2018; Griscom et al., 2017; Houghton & Nassikas, 2018). In this context, mechanistic models that quantitatively simulate long‐term C stocks and fluxes in forest ecosystems and promote the understanding of drivers of the forest transition are needed.

Two types of models for reconstructing long‐term C budgets in forest ecosystems are commonly used to assess forest C dynamics: (a) Simple bookkeeping models isolate the effect of wood extraction on forest C based on data on wood extraction, initial C stocks per unit area, and forest area (e.g., Böttcher, Freibauer, Obersteiner, & Schulze, 2008; Ciais et al., 2008; Hansis, Davis, & Pongratz, 2015; Houghton et al., 1983; Houghton & Nassikas, 2017). These models focus on the effects of wood extraction and, by factoring out changes in environmental conditions, are particularly valuable for quantifying land use impacts on C fluxes (Erb et al., 2013; Grassi et al., 2018; Le Quéré et al., 2015). (b) More complex process models are based on a set of equations describing the main eco‐physiological processes involved in plant C metabolism. These models explicitly account for pedoclimatic conditions and allow to quantify actual changes in ecosystem C stocks, including forests (e.g., Bellassen et al., 2011; Harrison, Jones, & Hughes, 2008; Schaphoff et al., 2018; Zaehle et al., 2006). However, while many process‐based models consider forest management, albeit implemented at varying levels of detail, secondary forest uses such as forest grazing, litter raking, pollarding, or pruning, which are key for the C state of ecosystems (Gimmi, Bürgi, & Stuber, 2008; Gimmi et al., 2013; Glatzel, 1991; McGrath et al., 2015) are usually omitted as a constraining variable (Le Quéré et al., 2015; Pongratz et al., 2018). This is a particularly important gap for analyzing long‐term C dynamics because these uses have been widespread, providing critical resources to pre‐industrial societies (Emanuelsson, 2009; Erb et al., 2013), and continue to greatly influence C‐dynamics at the global scale (Erb et al., 2018). Furthermore, due to the manifold processes accounted for and the prevailing knowledge gaps (Pongratz et al., 2018), simulations by individual process‐based models provide a massive range of outputs, thus indicating significant uncertainties that still prevail (Le Quéré et al., 2015). However, current bookkeeping models also show high variabilities in C emission estimates due to uncertainties driven by initial C stocks and temporal attribution of emissions from land use changes (Hansis et al., 2015; Pongratz et al., 2018).

This study presents a new modeling approach, CRAFT (CaRbon Accumulation in ForesTs), that enables to quantify the actual long‐term C budget in managed forests and better understand the drivers of forest transitions. The model focuses on the quantification of stand‐level forest C changes due to growth and mortality, environmental changes, and land management, while enabling the attribution of observed changes to underlying drivers at regional and national scales. One of the model's key aims is to depict complex ecosystem processes such as C stock changes as functions of C in‐ and outfluxes, and thus indirectly key dynamics such as age structure changes, while relying on readily available data on forest area and wood harvest and its replicability across scales. The approach thus combines the benefits of both bookkeeping and process models at intermediate complexity levels. We use long‐term (1850–2015) national and regional‐scale data for France to develop and validate the modeling approach and assess the drivers of the French forest transition through a decomposition analysis (Ang, 2005). The case of France is particularly useful for such an analysis because data on forest extraction and area are available at the regional level since the mid‐19th century, which corresponds to the beginning of the forest transition in France (Mather, Fairbairn, & Needle, 1999). Splitting the national level, which is the most commonly used spatial entity in forest transitions analysis, into a mosaic of regional dynamics provides a basis for better understanding of the diverse ecological conditions and management practices within the country and enables better parameterization and validation of the model. Our study seeks to answer two related questions: (a) Does the CRAFT model perform well in simulating available observed values of forest biomass and soil organic C (SOC)? (b) Which processes drive the French forest transition and its temporal dynamics?

## 2 METHOD

### 2.1 Design of the CRAFT model

The CRAFT model developed in this study aims at reconstructing long‐term C dynamics in forest ecosystems at the territorial and national scales. It simulates changes in C fluxes and stocks in biomass and soils of forested areas over periods of decades to centuries, based on information on forested areas, wood extraction, relation between net primary production (NPP) and biomass typical for the forest ecosystem, and pedoclimatic variables.

**Figure 1:**
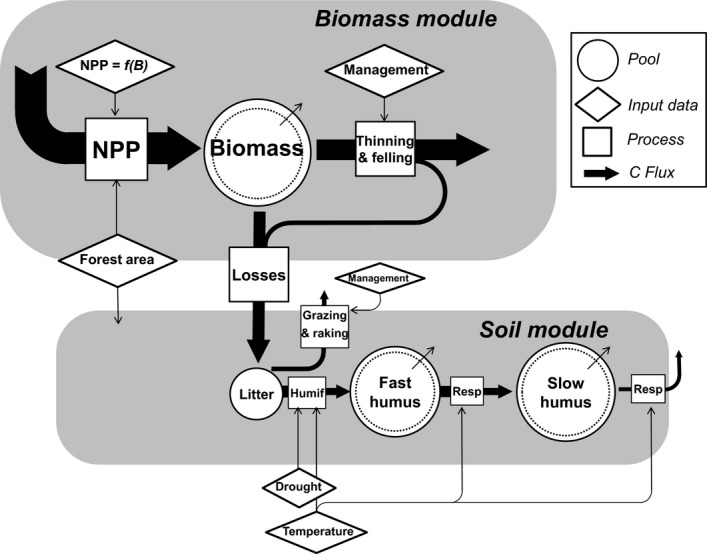
Basic structure of the CRAFT model. Central in the biomass module is the use of ecozone‐specific logistic relationships between net primary production and standing biomass derived from forest production tables (see text). The biomass module is linked to the soil module through biomass losses to litter following natural mortality and harvest. The soil module is based on the FORCLIM‐D model (Liski et al., 2002; Perruchoud et al., 1999)

The basic structure of the model is depicted in Figure 1. It is composed of two interconnected modules: a biomass module and a soil module. The former is based on the re‐interpretation of forest production tables, following an original procedure inspired by Duvigneaud (1971). The latter essentially follows the lines of the FORCLIM‐D model developed by Perruchoud, Joos, Fischlin, Hajdas, and Bonani (1999) and Liski, Perruchoud, and Karjalainen (2002). A user‐friendly guide of the model is provided in Data S1.

### 2.2 Biomass module

The biomass module uses annual data on forest area and wood extraction as input data, discerning deciduous and coniferous trees. The calculation in this module is based on an empirical relationship between NPP of trees and standing biomass stocks for the entire forest area of a given spatial unit (regional or national), distinguishing between deciduous and coniferous forests. The parameterization of this relationship is based on the data available in technical wood production tables established for each region and tree species by State Forest Services (Duvigneaud, 1971). Here, we used the *Tables de production pour les forêts françaises* by Vannière (1984). The production tables report data on standing volume and volume increment of the most important tree species in France, that is, discerning 13 coniferous and three deciduous tree species in different climatic regions in France (mainly oak, beech, pines, spruce, fir). Forest production tables provide curves of accumulated standing volume and annual increment of volume of exploitable wood as a function of age for monospecific tree stands. They generally distinguish three to five productivity classes, corresponding to different edaphic conditions in the region considered. The relationship established in this way applies to forest stands of one homogeneous age class. However, regional forests represent a mosaic of stands of differing ages. We considered that, if forests at regional or national scales are exploited at an average rotation time *rt*, the territory consists of *rt* age classes of similar size. We thus calculate the regional average standing stock and annual increment as a function of rotation time (see Data S3 for further details).

In order to establish the relationship between NPP and total standing biomass, we translated the variables reported in forest production tables, which refer to stem wood and timber only, into total C stocks and fluxes in forests. All stocks and fluxes are expressed in units of C, applying a wood C density factor of 0.47 to convert dry matter biomass to C stocks and fluxes (Eggleston, Buendia, Miwa, Ngara, & Tanabe, 2006). Wood volume was converted into tons C by applying factors for density (ton dry matter/m^3^) and C content (ton C/ton dry matter) of deciduous and coniferous trees of temperate regions as provided by the Eggleston et al. (2006). Total standing C biomass per hectare (biomass density) was subsequently calculated from wood standing stock (including stem and large branches) by adding the proportion of leaves, small branches, and roots as provided by Liski et al. (2002). Similarly, annual total NPP in ton C ha^−1^ year^−1^ was calculated from current annual wood production reported in production tables by adding the increment of leaves, small branches, and roots, using average data on the turnover time of these different compartments, based on Liski et al. (2002). Hence, the proportion of these tree parts to total tree C biomass and their turnover times were included in the calculation. The relationship between NPP and total standing biomass for a given tree species at the regional scale was fitted by a logistic function:

(1) $$\text{NPP}=rB*\left(1-B/K\right),$$

where NPP is the net primary production (ton C ha^−1^ year^−1^), *B* is the standing biomass (ton C/ha), *r* is the intrinsic growth rate (year^−1^), and *K* is the carrying capacity (ton C/ha).

Once the relationship between NPP and standing biomass was established, the gross annual increment in biomass (GAI; as ton C ha^−1^ year^−1^) of a forest at the regional scale can be calculated as the balance between NPP, natural mortality, and harvest rate *H* (ton ha^−1^ year^−1^). This dynamic is described by the equation:

(2) $$\text{GAI}=rB*\left(1-B/K\right)-\Sigma k_{i}*\varepsilon_{i}*B-H,$$

where *B* is the standing biomass (ton C/ha), *r* is the intrinsic growth rate (year^−1^), *K* is the carrying capacity (ton C/ha), *ε_i_* and *k_i_* represent the respective proportion to total biomass and turnover rates (year^−1^) of the different compartments (*i*, for leaves, small and large branches, stem, large and fine roots), as provided by Liski et al. (2002). Based on Equations (1) and (2), the key ecological processes annual NPP, GAI, and the annual growth rate (*r*) can be inferred. GAI is the core of the vegetation C sink as it refers to the fraction of NPP which survives at least one winter.

At the regional scale, we assumed that tree growth rates can be approximated by modeling the C‐dynamics of the most representative species of coniferous and deciduous trees. This enabled us to account for C‐dynamics due to shifts between deciduous and coniferous forests. We used the inventory data of the year 1985 provided by the IGN (2018) at the NUTS 3 level to select the three to four dominant coniferous and deciduous species of each regional unit considered here, which represented more than 80% of all tree species in most regions. Based on cross‐checks with figures on dominant tree species in terms of area in 1878 and 1908, we assumed that the dominance patterns of deciduous and coniferous assemblages were stable throughout the period. The regional *r* and *K* coefficients were then calculated as the weighted averages of these most representative coniferous and deciduous trees, respectively. The same procedure was applied at the national level, pooling the 16 different species reported for 1985. As statistical yearbooks do not provide information regarding the distribution of productivity classes of each tree among the total forest area, we fitted for each region the best productivity classes distribution for calculating standing biomass to empirical inventories data reported by the IGN (2018) for 1985, 1998, and 2011 (before 1985, no data on standing biomass were available at the regional level). These calculations were made using Microsoft Excel and associated VBA macros.

The *r* and *K* parameters derived from the production tables correspond to the pedoclimatic and management conditions in the 1960s, when these tables were established. A significant increase in tree growth rates occurred since the beginning of the 20th century in many regions of the Northern hemisphere (Boisvenue & Running, 2006; Gold, Korotkov, & Sasse, 2006; Köhl et al., 2015; Pretzsch, Biber, Schütze, Uhl, & Rötzer, 2014), including France (Badeau et al., 1996; Becker, 1989; Becker, Bert, Bouchon, Picard, & Ulrich, 1994; Becker, Niemen, & Gérémia, 1994; Charru, Seynave, Hervé, Bertrand, & Bontemps, 2017; Rathgeber, Guiot, Roche, & Tessier, 1999). This increase has been attributed to climate change (Girardin et al., 2011), particularly CO_2_ fertilization (Hickler et al., 2008), increased N deposition (Butterbach‐Bahl & Gundersen, 2011), genomic selection (Resende et al., 2012), or the combination of all these factors (Oren et al., 2001). In addition, the effect of forest secondary uses, for example, forest grazing or litter raking (Erb et al., 2013) changed throughout the period, contributing to forest degradation in the earlier parts of the investigation period and recovery in later phases.

In order to account for this observed long‐term increase of forest NPP since the last century, we assumed that the *r* and *K* parameters may have varied through time. To quantify the extent of their change, we first assumed that possible *r* and *K* parameter values in 1850 and 2015 ranged between 50% and 150% of the 1960 values. By assuming the same linear increases before and after 1960 for *r* and *K*, we calculated all possible combinations at the national level in the range considered (using discrete steps of 5%), fitting the results to empirical inventory data of both coniferous and deciduous biomass stocks reported by the IGN for 1985, 1998, and 2011. The assumption of linear increase of the *r* and *K* parameters is a simplification but in line with other studies (Ballantyne et al., 2017; Piao et al., 2018), which observed no significant changes in the rate of NPP increase during the 1960–2015 period.

The data on area and forest biomass harvest volumes were taken from forestry statistics at the departmental level (NUTS 3), separately for deciduous and coniferous tree species. For France, long‐term chronicles were reconstructed from the archives of the *Bibliothèque historique du Ministère de l’Agriculture* (http://www.unicaen.fr/mrsh/bibagri2/statistiques) for the years 1862, 1878, 1882, 1892, 1908, 1910, 1915, 1920, 1925, 1929, 1935, 1940, 1945, 1950, 1955, and 1960 and from the statistical yearbooks provided by Agreste (2019) and the IGN (2018) from 1947 to 2015. Harvest figures provided by statistics refer only to extracted commercial wood (stems and branches). Corresponding losses of non‐exploited organs (leaves, small branches, large and fine roots) left on the forest floor are also considered, applying the same data and assumptions as those made for the conversion of data in the wood production tables (Liski et al., 2002; Nabuurs et al., 2003; see also Data S1 for coefficient tables). For some years, only the total forest area and wood harvest were provided and the distribution between deciduous and coniferous was not reported. In those cases, we interpolated the proportion of deciduous and coniferous forest area and/or harvest from the two closest dates where these distinctions were made and applied to the total forest area and/or wood harvest. As the model is run on yearly steps, we interpolated the area and harvest between the two closest documented dates for each region. More information on data availability and interpolation is provided in Data S1. Biomass C stocks in new forest areas are assumed to be zero. Consequently, reforested areas contribute to decreasing biomass C density in the first years. However, if harvest of the total forest area remains constant, then increased forest areas lead to a drop in average yields, which may result in a rise in forests C stocks. The standing biomass in 1850 was initialized assuming that standing biomass was at equilibrium in 1850, that is, that NPP compensated losses through natural mortality and anthropogenic extraction. This assumption implies that the harvest rates reported in 1862 are reasonably representative for the preceding centuries. As no empirical information is available to validate this assumption, we performed a sensitivity analysis to test its influence (see below).

### 2.3 Soil module

Following the FORCLIM‐D model (Liski et al., 2002; Perruchoud et al., 1999, four compartments of litter were considered: coarse woody litter, large roots and branches litter, fine root litter, and foliage litter. These compartments were fed by natural losses from the different tree compartments, as well as by material left on the forest floor after felling or cutting. Each of these litter compartments is decaying at a specific rate. As mentioned above, natural transfers of standing biomass to litter are calculated using the proportion of each organ in total biomass and their turnover rates provided by Liski et al. (2002). Losses to litter by felling and cutting correspond to the parts of trees that are not extracted at harvest but left on the forest floor in standard harvesting practices. Losses at felling and cutting of different organs are calculated in proportion of extracted wood (as a proportion of each organ in total tree biomass), except for branches for which a ratio of non‐harvested to harvested material of 10% is assumed.

The decay rate of foliage and fine roots litter, the most reactive compartments of litter, is dependent on temperature and moisture: Liski et al. (2002) have fitted the following relationship to the data collected by Berg et al. (1993) with annual mean temperature and the difference between precipitation and potential evapotranspiration (PET) from May to September as explanatory variables:

(3) $$\begin{array}{cc} k_{\text{foliage}\;\text{and}\;\text{fine}\;\text{roots}\;\text{litter}}= & 0.35*\text{resp}*0.25\\ & \;*\left(1+0.094*\left[T-4\right]+0.0023*\left[M+50\right]\right), \end{array}$$

with *T* being the annual average temperature (°C) and *M* the difference between precipitation and PET from May to September (mm).

In historical periods and in some regions, a significant fraction of litter (mostly foliage, small branches, and brigs) was subject to harvest or grazing, as the forest was closely associated with the agricultural system (Gimmi & Bürgi, 2007; McGrath et al., 2015). A rate of harvest, up to 50% of the foliage litter production until World War II, was considered by Perruchoud et al. (1999) to fall under this category, based on data from Bürgi (1998). Here we applied these factors to all French regions from 1850 to 1940 and then assumed a gradual reduction of litter harvest and grazing until the end of the 1970s down to 0%. The sensitivity of the model to such grazing intensities in previous periods was evaluated through a sensitivity analysis (see below).

SOC stocks were divided into two humus compartments (Figure 1), differing by their turnover rates. The largest part of litter is released to the atmosphere as CO_2_, while a fraction is transferred to the fast humus compartment. The fast humus decay rate (*a*, year^−1^) is dependent on mean annual temperature (*T*) as a sole climatic control according to the relationship proposed by Trumbore, Chadwick, and Amundson (1996).

The slow humus compartment is characterized by a decay rate of 0.007 times the decay rate of the fast humus compartment (Liski et al., 2002; Perruchoud et al., 1999). This slow humus is fed by a fraction 0.0033 of fast humus decay. Given the value of these parameters, the steady state ratio of slow to fast humus is expected to be 0.007/0.0033 = 2.1. The initial stock of litter and humus was calculated at the equilibrium in 1850, that is, assuming that C inputs from biomass to soil and litter compensated losses through mineralization and transfer to other pools.

The two forcing climate variables, namely mean annual temperature (affecting mineralization rates) and potential evapotranspiration between May and September (affecting foliage and small root litter), were calculated for the different regions of France over the period 1990–2010 from the MESAN database, a European high‐resolution surface reanalysis (Häggmark, Ivarsson, Gollvik, & Olofsson, 2000; Landelius, Dahlgren, Gollvik, Jansson, & Olsson, 2016). Potential evapotranspiration (PET) has been calculated using the formula proposed by Oudin et al. (2005):

(4) $$\text{PET}\left(T\right)=\left(T+5\right)*\text{Re}/l*r*100,$$

with PET being the potential evapotranspiration in mm/day, *T* the temperature in °C, Re(*t*) the extraterrestrial radiation in MJ m^−2^ day^−1^, *l* the latent heat of vaporization in MJ/kg, and *r* the water density, that is, 1,000 kg/m^3^.

To calculate these forcing climate variables for the period 1850–1990, we applied the approach developed by Le Noë, Billen, Mary, and Garnier (2019), based on available data of the temperature anomaly in the period 1990–2010. The above formula is used to calculate PET as a function of this temperature anomaly. In the absence of evidence for significant variation, interannual average pluviometry is considered constant over the whole period, as assumed by Garnier et al. (2019).

The land‐use change from agricultural land (cropland and grassland) to forest area that may have occurred from 1850 to 2015 was accounted for if, in a given year, agricultural land declined while forest land expanded, considering only net land conversion (Fuchs et al., 2016). The fast and slow humus content in agricultural soils in each French region from 1850 to 2015 was derived from previous works by Le Noë, Billen, Mary, et al. (2019) and Le Noë, Billen, and Garnier (2019). Biomass and litter content of agricultural soils were neglected. For each time step from year *n* to *n* + 1, the fraction *ɛ* of forest area which was converted from agricultural land was calculated, and the forest per area stock of each compartment (C*_F_*) was corrected according to the formula:

(5) $$C_{F\;n+1}=\left(C_{F\;n}+\Delta C_{F\;n}\right)*\frac{1}{1+\varepsilon }+\left(C_{A\;n}\right)*\frac{\varepsilon }{1+\varepsilon },$$

where C*_A n_* is the corresponding per area stock of C (biomass, litter, fast and slow humus) in agricultural land at year *n*. The values of *ε* are generally close to zero and reach a few percent only during limited periods in specific regions. Considering land use changes thus introduces only a second‐order correction during these periods. Note that if a land‐use change occurs and impacts forest SOC, this will be translated on a per hectare basis as a *dilution* of SOC stocks due to the inputs of SOC from grassland and cropland which generally have lower SOC contents.

### 2.4 Decomposition analysis

We conducted a decomposition analysis to quantify the major biophysical drivers of forest biomass C stock change, following the idea of a forest identity analysis (Kauppi et al., 2006). Here, we distinguished as potential driving forces the total forest area (*A*), the composition of coniferous and deciduous areas (SC*_i_*), and C density in coniferous and deciduous biomass (*D_i_*), which reflected the productivity increase in relation to harvest intensity and *r* and *K* change:

(6) $$B=\sum_{i}A*\frac{A_{i}}{A}*\frac{B_{i}}{A_{i}}=\sum_{i}A*{\text{SC}}_{i}*D_{i}.$$

An additive decomposition analysis using the Logarithmic Media Divide Index (LMDI) proposed by Ang (2005) was conducted at the national level to quantify the relative contribution of *A*, SC, and *D*:

(7) ΔB=ΔA+ΔSC+ΔD,

with Δ*B*, Δ*A*, ΔSC, and Δ*D* being, respectively, the changes in total biomass C stocks (M ton C), changes in area (Mha), changes in species composition (ratio of coniferous and deciduous areas to total forest area), and changes in biomass density (M ton C/Mha).

### 2.5 Uncertainties and model validation

The final results of standing biomass and SOC content in forests in French regions derived by CRAFT were compared with current existing data on standing biomass for the years 1985, 1998, and 2011 (IGN, 2018) and forest SOC content in the 2010s as collected by the Network of Measurements and Quality of Soil (RMQS). In addition, the CRAFT model results were also compared with model results from a widely used bookkeeping model (“Houghton model”) for land‐use induced C emissions (Houghton, 2003; Houghton et al., 1983; Houghton & Nassikas, 2017; Le Quéré et al., 2015). We used a matlab version (Erb et al., 2013) of this model for an analysis at the national level for the period 1850–2015. Contrary to the CRAFT model that simulates forest C stocks, the Houghton model isolates the effects of wood extraction and forest area changes on forest biomass C stocks (Houthon, 2003). Note that this model neither depicts the effects of environmental changes induced C‐dynamics nor those of forest secondary uses. Therefore, in order to warrant comparability, static *r* and *K* parameters were used in a control‐run with CRAFT, that is, simulating constant forest growth conditions. We called this run “static CRAFT” to distinguish it from the standard CRAFT which uses dynamic parameters. Fluxes related to wood products were excluded in both models, the bookkeeping model and static CRAFT.

Because the outputs of the simulation depend on some major assumptions in the parameterization and the structure of the model, a sensitivity analysis was performed at the national level. The sensitivity of results to variations in the following input data was tested: (a) the distribution of forested areas among productivity classes, (b) the initialization of standing biomass in 1850, (c) the intensity of litter harvest and grazing in historical periods.

## 3 RESULTS

### 3.1 National trends and alignment with other assessments

**Figure 2:**
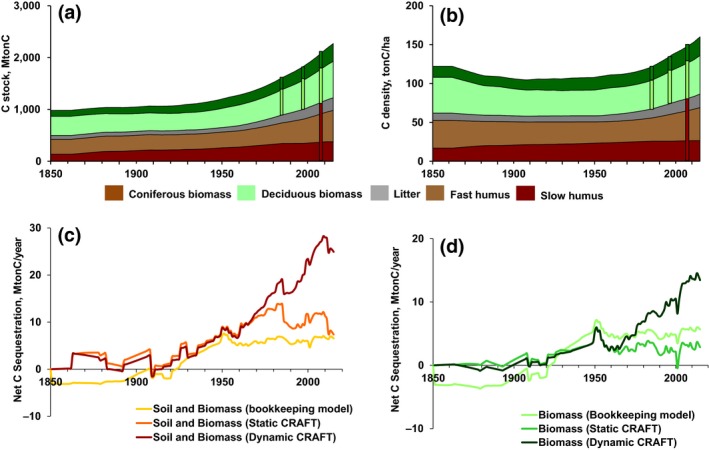
C stocks (a) and C density (b) in forest ecosystems in France from 1850 to 2015 as simulated by the CRAFT model (continuous lines) and as observed for some recent years (bars) by the IGN (2019) and RMQS. These simulations are obtained by considering France as a single region (see Data S2 for details on calculation). Net C sequestration in forest ecosystems including soil and biomass (c) and only forest biomass (d) in France as simulated by the bookkeeping model by Houghton; the static CRAFT (constant *r* and *K* parameters) and the CRAFT (dynamic time‐dependent *r* and *K* parameters)

The CRAFT model result shows a continuous increase by 130% in total C stocks in French forests between 1850 and 2015 (Figure 2a). SOC and biomass stocks increased equally, with SOC contributing c. 51%–55% to total stocks throughout the period. The distribution among coniferous and deciduous trees changed only marginally, with the fraction of coniferous biomass stocks increasing from 23% to 33% of total biomass stocks between 1850 and 2015. C density, that is, C stock per unit forest area, followed a different trajectory. C density (particularly in biomass) decreased in the late 19th century, stagnated from c. 1900 to 1950, and increased again in recent decades due to trends in both SOC and biomass (Figure 2b).

The model shows a good performance in comparison with the soil carbon and biomass stocks as reported by forest inventories (*p* < .05), in particular related to the biomass module, for example, in terms of total stocks (M ton C), biomass density (ton C/ha), and variation of total stocks (∆M ton C 1985–2011; Figure 2a,b; see Data S1 for additional figures). This good performance was enabled by the calibration of the evolution of *r* (growth rate) and *K* (carrying capacity) parameters and the optimization of productivity classes distribution. The best fit was found with the combination of increasing *r* and *K* by 15% for coniferous and no change for deciduous in the first period (1850–1960), and an increase by 35% for coniferous and 15% for deciduous species in the second period (1960–2015) and with productivity classes distribution according to the ratios 0:0:1:4 and 0:1:1:8 for deciduous and coniferous, respectively. Note that only permutations in these two periods and only linear changes of *r* and *K* were assumed, which nevertheless yields a remarkably good fit. This optimization of the *r* and *K* parameters is consistent with findings from previous studies highlighting that significant increase of growth rates and carrying capacities occurred since the 1960s (Pretzsch et al., 2014), while gentler increase in growth rates likely happened before the 1960s due to changing land‐use practices and improving species selection (Erb et al., 2013).

The sensitivity of the model toward productivity class distribution was also explored with productivity classes distributed according to the ratios 1:1:1:1 and 1:2:2:1, instead of calibrating this distribution. Both scenarios led to higher estimations of C stocks in biomass (from 34% to 55%), both in 1850 and 2015 but decreased correlation with observed data. This simulation indicates that optimization of productivity classes distribution is key for simulating reliable outputs (see Data S1 for additional figures on sensitivity analysis).

Outputs simulated by the CRAFT model for France were compared to a simulation over the same study period (1850–2015) using the bookkeeping model by Houghton et al. (2000; Figure 2c,d). According to the bookkeeping model, wood extraction initially induced a C source from forest biomass which peaked in the late 19th century and then decreased. Forests turned to a C sink around 1930 (Figure 2d), peaking in 1950, and after a decline stagnated from 1960 onward. In contrast, the static (constant *r* and *K* parameters) and standard (dynamic time‐dependent *r* and *K* parameters) CRAFT simulations hold that biomass in forests acted as a C sink from 1900, and presented some oscillations between source and sink before 1900 (Figure 2d). From 1950 onward, the static CRAFT model results are closely in line with the bookkeeping model, in particular related to the trends and to a lesser degree, related to absolute values (Figure 2d). By contrast, the decline of the biomass sink in the bookkeeping model in the decades after 1950 is not reproduced by the standard CRAFT, which shows an increasing C sink in biomass. Despite the apparent increasing differences between CRAFT, static CRAFT, and the bookkeeping model after 1960 (Figure 2d), there was no acceleration of the divergence in C sequestration. In the end, over the 2000–2015 period, the static CRAFT and bookkeeping models led to average GAI in biomass five and six times lower, respectively, than the dynamic CRAFT simulation. These differences represented average net annual C sequestration in biomass of 2.1, 2.7, and 13 M ton C/year by the bookkeeping model, static CRAFT and CRAFT, respectively.

At the national level, the model estimated an average SOC content of 70 ton C/ha, in good agreement with the average measured value of the RMQS (77 ton C/ha). Although only poor correlation was found at the regional scale, SOC values obtained by the CRAFT model in 2015 for the different regions were in good agreement with measured data from the RMQS: c. 75% of calculated values fall in a range from 75% to 125% of measured values. SOC estimates by the model were never 60% lower or higher than measured ones, while measurements from the RMQS are likely to be subject to high spatial and temporal variability (Conant, Smith, & Paustian, 2003; Davidson, Belk, & Boones, 1998; Jandl et al., 2014). The significant discrepancies between measured and simulated SOC stocks at the regional scale may be explained in part by the uncertainty on the initial SOC value. Similarly, the model developed by Liski et al. (2002) has been shown to be highly sensitive to the initialization step (Peltoniemi, Palosuo, Monni, & Mäkipää, 2006). Here, the initial SOC was defined as the steady‐state SOC value for the management and climatic conditions in 1850; this steady‐state hypothesis seems reasonable when averaged at the national scale of France but might be more questionable when downscaled to the regional level. The sensitivity analysis explorations of lower and higher initial standing biomass values influenced C stocks both in biomass and SOC, particularly in the first 50 years of the simulation. However, the relative gap diminished significantly over time and reached only 1%–3% in 2015, thus suggesting that the standard hypothesis of steady state in 1850 is acceptable.

Another source of uncertainties regarding the estimation of SOC stocks is the assumption made about litter extraction through harvest and grazing in historical periods. At the national level, this hypothesis was tested by assuming, besides the standard hypothesis of 50% of annual litter production until 1940, two scenarios with no litter extraction and 100% litter extraction, respectively. The results showed SOC values 75% higher and 75% lower with respect to the standard hypothesis in 1850. However, this relative gap was much lower in 2015, reaching SOC stocks merely 23% lower and higher in the scenario with no litter grazing and 100% of litter grazing, respectively. Our standard hypothesis provided SOC estimations closer to the measured values than did the two scenarios, thus suggesting that this hypothesis is more realistic than the other two. However, as for the initialization step, this assumption might be more suitable at the national level but associated with higher uncertainties at the regional level due to heterogeneous secondary uses of forest at the regional level. Comparison with the bookkeeping model revealed that the relative contribution of soils to C sequestration was more significant in both CRAFT simulations than in the bookkeeping simulation (Figure 2c). The uncertain assessments of trends in SOC stocks confirm the high uncertainty of this C compartment (Jandl et al., 2014) compared to more robust assessments of biomass C stocks.

### 3.2 Regional results

**Figure 3:**
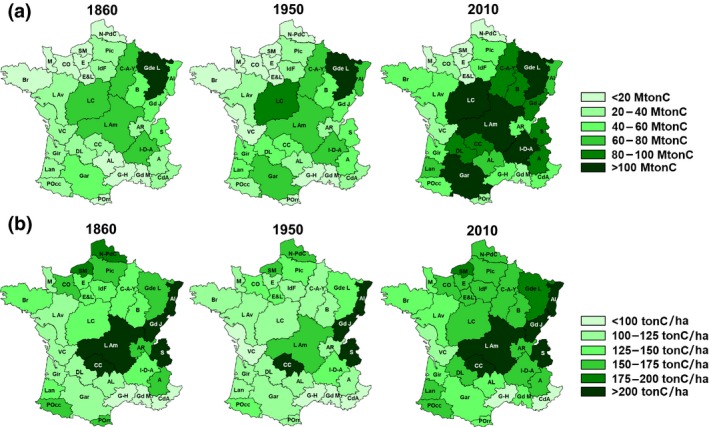
Total C stocks (a) and density of C stocks (b) in forest ecosystems, including both biomass and soil, in 1860, 1950, and 2010*.* A, Alpes; Al, Alsace; AL, Aveyron‐Lozère; AR, Ain‐Rhône; B, Bourgogne; Br, Bretagne; C‐A‐Y, Champagne‐Ardennes‐Yonne; CC, Cantal‐Corrèze; CdA, Côte d’Azur; CO, Calvados‐Orne; DL, Dordogne‐Lot; E, Eure; E&L, Eure‐et‐Loire; Gar, Garonne; Gd J, Grand Jura; Gd M, Grand Marseille; Gde L, Grande Lorraine; G‐H, Gard‐Hérault; Gir, Gironde; I‐D‐A, Isère‐Drôme‐Ardèche; IdF, Ile de France; L Am, Loire Amont; L Av, Loire Aval; Lan, Landes; LC, Loire Centrale; M, Manche; N‐PdC, Nord Pas‐de‐Calais; Pic, Picardie; Pocc, Pyrénées Occidentales; POr, Pyrénées Orientales; S, Savoie; VC, Vendée‐Charentes

The national trends are the combined effects of regional trajectories of very high spatial heterogeneity, which were computed individually for each of the 33 regions in France. Figure 3 displays forest C stocks and densities at the regional scale in 1850, 1960, and 2010. Differences in C stocks on this spatial level are not only the result of a strong heterogeneity in forest area between regions but also in forest C density (varying from 52 to 285, 62 to 220, and 93 to 283 ton C/ha among regions in 1860, 1950, and 2010, respectively). This indicates that regional disparities in forest management and edapho‐climatic conditions strongly influence the level of C stocks in forest.

In spite of these disparities, in most French regions, forest C stocks increased from 1850 onward. C sequestration in forests corresponds to increased C stocks in standing biomass (Figure 3a), litter, and SOC. The most significant increase occurred from 1950 to 2010, resulting from both forest area expansion and a significant increase in biomass density (Figure 3b). The slight shift toward more coniferous forests observed at the national scale owes mainly to management changes in the highly productive regions of Landes and Gironde in the South‐West of France which underwent a shift in species composition.

Even though there were large differences between individual regions, the results of the CRAFT model run for France as a whole, using average control variables, were very similar to those obtained as the sum of the 33 regions, thus showing very little scale effects (see Data S1). This indicates that the model is well suited to work both at the regional and national levels.

### 3.3 Drivers of biomass C stocks change during the forest transition

**Figure 4:**
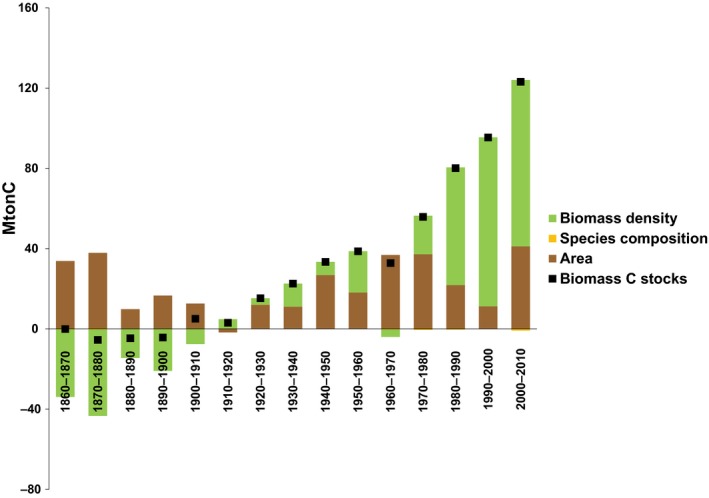
Decomposition analysis quantifying the contribution of area (*A*), species composition (SC), and biomass density (*D*) in the forest biomass C stocks (B) changes in France from 1860 to 2010. Note that the effect of species composition is too small to be displayed (see Data S2)

A decomposition analysis at the national level revealed clear temporal trends in the relative effect of different drivers on biomass C stocks dynamics (Figure 4).

The decomposition analysis shows that the French forest transition since the mid‐19th century was initially driven by area expansion. Shifts in species composition played a negligible role. However, although growing forest area throughout the whole period positively affected C sequestration, its relative contribution was variable. From 1860 to 1980, it was the main driver of increasing biomass C stocks, then change in biomass density became the predominant mechanism of C sequestration. Initially in the mid‐19th century, the change in density was negative, counteracting the positive contribution of expanding forest area. The decline in biomass density can be attributed to increased levels of wood harvest at the end of the 19th century and to the rejuvenation of standing biomass following area expansion. However, decreasing biomass density might be due to an overestimation of the initial standing stocks and an underestimation of the actual wood extraction in the mid‐19th century. It is indeed likely that some wood harvest by the rural population had not been recorded by the official national statistics since the *Code Forestier* implemented in 1827 aimed at restricting the access to forests for the rural population (Mather et al., 1999). A transition occurred around 1910 and since then, forest biomass thickening has increasingly amplified the sequestration of C in French forests. Increasing biomass density even became the predominant factor of C net sequestration in the last three decades.

## 4 DISCUSSION

In the present study, we developed the CRAFT model to analyze long‐term forest C dynamics and tested it for the case of France at the national and regional scales in the period 1850–2015. Our empirical results highlight that the French forest transition fostered C sequestration in soils and biomass, particularly since the beginning of the 20th century, although forest area had already increased since at least the mid‐19th century. Two major factors were shown to foster these changes: forest area and biomass density. In contrast to other case studies (e.g., Austria, Gingrich, Erb, Krausmann, Gaube, & Haberl, 2007; USA, Magerl et al., 2019), change in forest area was the predominant driver until the 1970s. One open question that still remains is to elucidate the different processes that influenced the observed changes in forest area and biomass density.

### 4.1 Drivers of forest expansion

In the late 19th and early 20th centuries, the expansion of forest area was made possible by agricultural intensification, notably the development of fodder crops and abandonment of extensive grazing and other marginal land uses (Erb et al., 2008; Gingrich et al., 2019; Mather & Needle, 1998), and through the development of international agricultural trade promoted by the free trade agreements under Napoléon III (Duby & Walon, 1993). However, new regulations and rules governing the access and use of forests also impacted forest change, as did a more acute perception of forest degradation and the threat that it represents for the French economy (Mather et al., 1999). After WWII, the Monnet plan (1945), the Marshall plan (1947) and, later, the creation of the Common Agricultural Policy (1962) engendered tremendous intensification and specialization of the agricultural sector (Le Noë, Billen, Esculier, & Garnier, 2018; Le Noë, Billen, Mary, et al., 2019). Mechanization based on fossil fuels and the increase of the average farm size promoted rural exodus and a shrink in agricultural land, which concentrated on the best soils, freeing space for forest expansion. Concomitantly to the abandonment of agricultural land, several incentive policies in favor of afforestation have been implemented since WWII, including the creation of a national fund for forestry in 1946 and the modernization of institutions, essentially via the creation of the National Forestry Office in 1964 fostering afforestation projects (Corvol‐Desset, 2016).

However, the transformation of agriculture which rendered the forest transition in France possible came at a high environmental cost in terms of greenhouse gas emissions (Garnier et al., 2019; Le Noë, Billen, Mary, et al., 2019). Therefore, the estimation of average annual C sequestration in forest biomass and soils of 97 M tons CO_2_ eq./year (this study) and the figures of c. 6 M tons CO_2_ eq./year C sequestration in agricultural land (Le Noë, Billen, Mary, et al., 2019) over the 2000–2010 period should be compared to the estimated emissions of the French agricultural sector of c. 114 M tons CO_2_ eq./year in 2010 (Garnier et al., 2019). Garnier et al. (2019) calculated that agricultural emissions in France in 1850 were only as high as c. 30 M tons CO_2_ eq./year. Therefore, agricultural intensification and specialization enabled C sequestration in the course of the forest transition, but was only possible by mechanization, inputs of mineral fertilizers, long‐distance trade of animal feed, and increasing livestock density, which in turn generated significant amounts of greenhouse gas emissions. Balancing the climate‐change mitigation effects of forest transitions against their “hidden emissions” (Gingrich et al., 2019) emphasizes the need to further integrate land‐use modeling tools, such as the CRAFT model, in order to better quantify and allocate the C budgets related to long‐term land‐use change.

### 4.2 Drivers of forest biomass thickening

The good alignment of the biomass density as estimated by the CRAFT model in its dynamic version with the inventory data indicates that it was more accurate than simulations by the static CRAFT; this points to the pivotal role of changes in growth parameters in the last centuries. Not surprisingly, CRAFT results differ from results of the bookkeeping model by Houghton, which is designed to isolate land use–related C fluxes and not to reconstruction carbon stocks in the series. Our results are very similar to the findings by Erb et al. (2013) for the case of Austrian forests, who also find an increasing divergence from the 1950s onward between the net C sink in forest biomass as measured by long‐term forest inventory data and the C source as estimated with the bookkeeping model by Houghton. The authors demonstrated by the mean of least‐square fit analyses that this divergence in the 1950s was enabled only because biomass density started to increase since the 1910s. They further hypothesized that this may have been due to change in forest secondary use. In contrast, other studies based on the comparison of two simulation sets by dynamic vegetation models, that is, with and without land‐use change, argue that enhanced C sequestration in forest biomass since the 1960s was only environmentally induced (Le Quéré et al., 2015). Our results can contribute to this debate as the comparison of the simulations by the CRAFT, static CRAFT, and bookkeeping model by Houghton can be also useful to isolate different drivers that affect the trajectories of biomass density, growth annual increment, growth rates, and carrying capacities.

From the mid‐19th century to end of the 1940s, differences between the CRAFT and static CRAFT are small while they are more significant with the bookkeeping model, both for biomass and biomass and soils (Figure 2c,d). The similarity of results from CRAFT and static CRAFT in this period suggests that changes in intrinsic growth rate were not the predominant factor controlling changes in biomass density. As the calculated changes in intrinsic growth rate concerned only coniferous stands in a time when climate change was still weak, these changes can more likely be attributed to changes in secondary uses of coniferous forests. This is also consistent with the fact that coniferous forests were always the most heavily exploited ones and were therefore more likely subject to changes in secondary uses. At the same time, while wood was the main fuel in France until the 1870s (Smil, 2017), providing energy for heating and cooking in French homes, the switch from wood to coal certainly relieved pressure on wood extraction and may have been the prime reason of the observed transition of forest biomass density (Figure 4). Therefore, another relevant factor to explain the divergence between the CRAFT and bookkeeping models is the effect of forest age structure, controlled by the expansion of forest area and variation in harvest rates, which affects the growth rate of forest stand density (He, Chen, Pan, Birdsey, & Kattge, 2012; Noormets et al., 2015; Pan, Birdsey, Phillips, & Jackson, 2013; Pretzsch et al., 2014; Pugh et al., 2019; Vilén et al., 2012). This effect of age structure can be accounted by both the CRAFT and static CRAFT approaches thanks to the nonlinear relationship between NPP and standing biomass. Indeed, while the dynamic assessment of tree age distribution is a tricky step of most process‐based models (Bellassen et al., 2011; Zaehle et al., 2006), the CRAFT model builds upon a simplified approach by deriving increment not mechanistically from tree‐age distribution, but statistically from standing biomass. The observed difference between static CRAFT and the bookkeeping model (Figure 2d) thus suggests that the evolution of forest age structure was the major process influencing changes in biomass density from the mid‐19th century to the 1940s. Similar to Erb et al. (2013), it can, therefore, be assumed that the observed thickening of biomass from 1910 was fostered by the recovery from an initial loss of biomass density in the second part of the 19th century, resulting in a stronger potential to sequester additional C in biomass, in a period where climate change can be assumed to have played a negligible role (Erb et al., 2013). This corroborates that using the bookkeeping approach is indeed likely to introduce a significant bias in the estimation of the impact of land use on C fluxes, by failing to account for the legacy of the past land use management and changes in forest secondary uses.

By contrast, since the 1960s, the simulation of the CRAFT model was increasingly divergent to the simulation by the static CRAFT and bookkeeping model, both for soil and biomass (Figure 2c,d). Conversely, the static CRAFT simulated values closer to the bookkeeping model for biomass (Figure 2d), but estimated higher sequestration in soils (Figure 2c). These comparisons suggest that changes in intrinsic growth were the predominant factor controlling changes in biomass density after 1960 (Figures 2b and 4). Changes in age structure only played a modest role, explaining the small difference between the static CRAFT and the bookkeeping model. However, uncertainties still remain regarding the factors that fostered changes in intrinsic growth rate in this period, as it could be both environmental conditions (Noormets et al., 2015) and forest secondary uses (Erb et al., 2013, 2018). Further development of the CRAFT model, in combination with other approaches, is required to disentangle the relative importance of changing environmental conditions and forest secondary uses after the 1950s to 1960s.

In summary, the rejuvenation of forests from the mid‐19th century to the mid‐20th century was certainly the major process controlling changes in biomass density. It resulted in an initial loss of forest biomass, the recovery from which led to C sequestration in biomass. Since the 1960s, change in intrinsic growth rates was the most important driver explaining the sharp improvement of gross annual C increment and biomass density.

## 5 CONCLUSIONS

The study results in major empirical and methodological conclusions.

- Methodologically, we have demonstrated that the CRAFT model enables reliable simulations of the dynamics of C stocks and fluxes during a forest transition, both at the regional and national levels. The model is able to depict actual C‐dynamics of forest stands resulting from the combination of environmental and management effects, and not, as most bookkeeping models, only account for the fluxes related to wood extraction. One of the main advantages of using the CRAFT model is that by using historically variable ratios of NPP to biomass volume as an input, it incorporates trends of increasing growth rates. Thus, it overcomes the general shortcoming in international data that empirical time series of forest increment are not available. As France encompasses very diverse pedoclimatic contexts, including temperate, continental, Mediterranean, and mountainous regions, we argue that the CRAFT model could be used for other countries with similar contexts, as far as input data are available. CRAFT could thus facilitate further investigations of national C dynamics in forests in long‐term research.
- Empirically, we conclude that the forest transition in France fostered C sequestration in forest biomass and soils from the mid‐19th century onward. Changes in tree species distributions were marginal and did not have major effects on changes in C sequestration. By contrast, the rate of expansion of forest area generated a large constant impact on C sequestration for as long as 160 years. There was an interruption only in the 1910s, coinciding with World War I, when the forest area of France ceased to expand. Also forest biomass density changed dramatically, which significantly affected C sequestration in forests. This change in biomass density resulted from the effects of changed secondary forest uses and age structure in early periods and additionally changing environmental conditions in later periods. The relative contributions of these drivers remain, however, unclear and should be addressed in future research.

## Data Availability

The data that support the findings of this study are all available in the supplementary materials of this article. See Data S1 for more detail information on the method, hypothesis, and regional parameters; see Data S2 and Data S3 for calculation procedure and regional input data.
