# Supplementary material for: Modeling and empirical validation of long‐term carbon sequestration in forests (France, 1850–2015)
Source: Glob Chang Biol. 2020 Feb 13;26(4):2421–34. doi: 10.1111/gcb.15004 (PMC7154705; doi:10.1111/gcb.15004)
Supplement: Supplementary file 1 [file GCB-26-2421-s001.docx]

**Supplementary Online Material (SOM1) to the paper:**

**Modelling and empirical validation of carbon stock accumulation during the forest transition in France 1850-2015**

Julia Le Noë^1^, Karl-heinz Erb^1^, Sarah Matej^1^, Andreas Magerl^1^ Manan Bhan^1^, Simone Gingrich^1^

^1^Institute of Social Ecology (SEC), Department of Economics and Social Sciences, University of Natural Resources and Life Sciences, Wien, Austria

**Methodological notes**

*This note contains an extensive description of the data compilation and hypotheses used for the development of the CRAFT (Carbon Reservoir Accretion in ForesT) model and its application to French case, a summary of which is provided in the material and methods section of the main article. This note ends with a short list of instructions for future users.*

1. **Introduction**

The CRAFT model (Carbon Reservoir Accretion in ForesT) is a simplified representation of carbon dynamics in forest ecosystems at the territorial scale over the long term. It aims at simulating changes in C fluxes and storage in biomass and soils of forested areas of countries over periods of decades to centuries, from chronicles of forested areas, wood extraction, climatic variables and other agricultural land use and productions.

The basic structure of the model is described in Fig. 1. It is composed of two interconnected modules: a Net Primary Production (NPP) module and a litter and soil organic matter module. The former is based on the re-interpretation of forest production tables, following an original procedure inspired from Duvigneaud et al (1971). The latter essentially follows the lines of the FORCLIM-D model developed by Perruchoud et al. (1999) and Liski et al (2002) and calibrated on ^14^C/^12^C ratios in litter and humus compartment in response to the transient signal of bomb produced ^14^C in the 1950-1963 period.

**
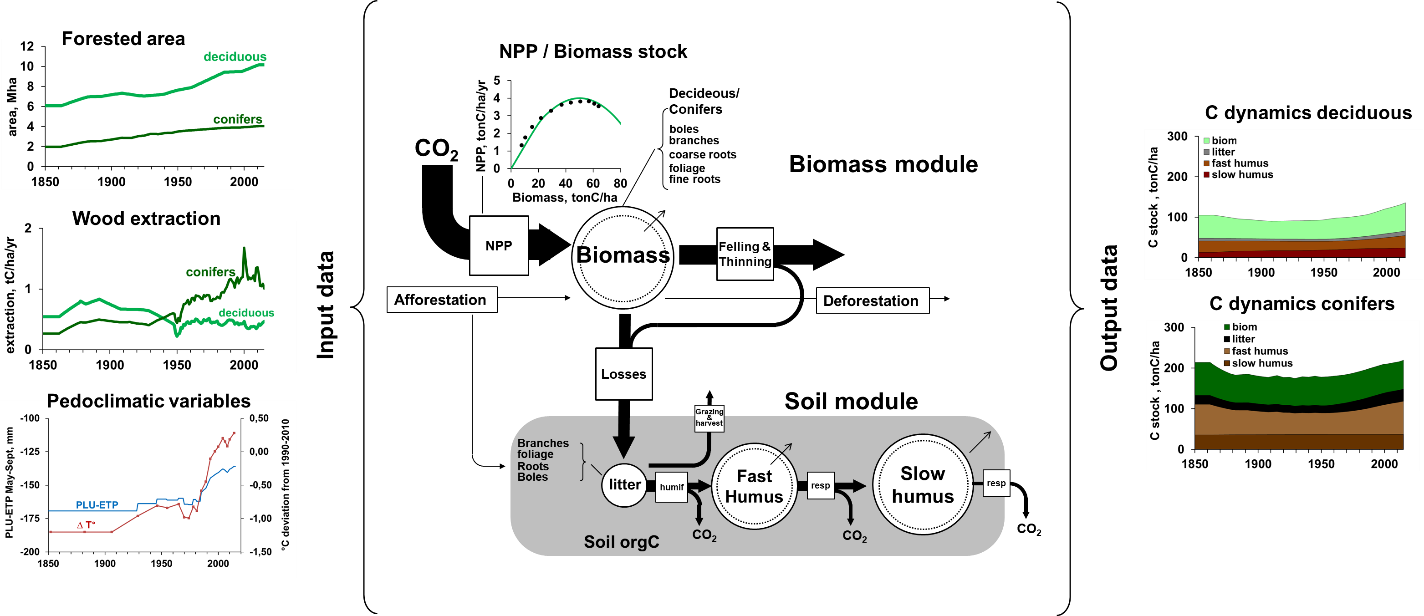
**

**Figure 1**. Basic structure of the CRAFT model. The biomass module uses chronicles of coniferous and deciduous surfaces and harvest rate as inputs data, the figures above are here provided for France (1850-2015). A logistics relationship between NPP and standing biomass is derived from yields table and serve for model parametrization. The distribution of productivity classes and the evolution in time of the equation parameters are optimized with a routine using Macro excels so that simulated data best fit to the available observed data. The biomass module is linked to the soil module through biomass losses to litter following natural mortality and harvest. The soil module is taken from the ForClimD model (Perruchoud et al., 1999; Liski et al., 2002). The only inputs data required for the soil module are the chronicles of average annual temperature and of drought from May to September, calculated as the difference between evapotranspiration and precipitation. SOC change following land use change are calculated based on previous assessments by Le Noë et al., (2019a, b) of SOC in agricultural land.

1. **The Net Primary production module**

The calculation in this module is based on an empirical relationship between NPP and standing biomass for the whole of the forest area of a given region, distinguishing however between deciduous and conifer forests. According to the classical procedures in use since the early work of forest ecologists (Duvigneaud et al., 1971), the parameterization of this relationship is based on the data available in technical wood production tables established for each region and tree species by State Forest Services. In particular, for the case of France, we used the *Tables de production pour les forêts françaises* by Vannière (1984).

**2.1. Wood production**

Forest production tables provide curves of cumulated standing stock and annual increment of exploitable wood as a function of age for monospecific trees stand. They generally distinguish a 3 to 5 productivity classes, corresponding to different edaphic conditions in the region considered. Because of the non-linearity of this relationship, the relationship established in this way does not apply to a mosaic of stands of differing ages as they exist at the regional scale. If it is considered that regional forests are exploited according to a rotation time rt, the territory can be considered as consisting of rt age classes of similar size, and the regional average standing stock and annual increase can be calculated, as a function of rotation time. A new relationship is obtained, as shown in the example of Fig. 2.

***Figure 2****. Relationship between wood production and wood standing stock established for single stands of increasing ages (direct data from technical wood production tables) and for a regional mosaic of stands exploited at different rotation times. Example case of beech in NE France, productivity class II.*

**2.2. Net primary production**

Wood volume were translated into ton C by applying the density (ton dry matter per m^3^) and C content (ton C per ton dry matter) of deciduous and coniferous trees of temperate regions as provided by the IPCC (2006). Total standing biomass can be calculated from wood standing stock (including stem and large branches) by adding the proportion of leaves, small branches and roots as provided by Table 1a.

Net primary production is calculated from wood production by adding the production of leaves, small branches and roots, calculated using average data on the turnover time of these different organs (Table 1b), taken from Liski et al (2002), providing the proportion of these organs to total tree C biomass and their turnover times.

**Table 1**. **a**. *Proportion of different organs to total C tree biomass (data from Liski et al 2002).*

|  |  | **deciduous** | **conifers** |
| --- | --- | --- | --- |
| stem | fraction | 0.67 | 0.60 |
| branches | fraction | 0.21 | 0.26 |
| large roots | fraction | 0.09 | 0.07 |
| fine roots | fraction | 0.01 | 0.01 |
| foliage | fraction | 0.01 | 0.05 |
| total |  | 1.00 | 1.00 |

**b.** *Turnover time of different organs (data from Liski et al., 2002)*

|  |  | **deciduous** | **conifers** |
| --- | --- | --- | --- |
| coarse woody litter | yr^-1^ | 0.0087 | 0.0043 |
| branches & large roots | yr^-1^ | 0.025 | 0.027 |
| fine roots | yr^-1^ | 0.87 | 0.87 |
| foliage | yr^-1^ | 1 | 0.2 |

A relationship can therefore be established between NPP and total standing biomass for a given tree species at regional scale, as shown by the example of Fig. 3.


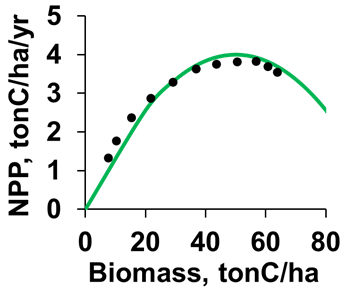


***Figure 3****. Empirical relationship between net primary production and total standing stock for a regional mosaic of stands of different ages exploited with different rotation times. Example case of Maritime Pine in the South-West of France, productivity class III. Comparison to a logistic relationship with parameters r = 0.12 yr^-1^ and K = 100 tonC/ha.*

This empirical relationship can be fitted by a logistic function of the form:

NPP = rB*(1-B/K) (1)

Where NPP is the net primary production (tonC/ha/yr)

B is the standing biomass (tonC/ha)

r is the intrinsic growth rate (yr^-1^)

K is a carrying capacity term (tonC/ha)

Table 2 gathers the values of parameters r and K obtained with this procedure for different tree species in different French regions.

**Table 2**. *Parameters of NPP for major tree species in different regions of France, calculated from Forest production tables (Vannière, 1984) r: intrinsic growth rate in yr-1; K carrying capacity in tonC/ha*

|  | **Class 1** | | **Class 2** | | **Class 3** | | **Class 4** | |
| --- | --- | --- | --- | --- | --- | --- | --- | --- |
|  | r | K | r | K | r | K | r | K |
| **Scotch Pine Sologne** | 0.11 | 200 | 0.11 | 150 | 0.08 | 150 |  |  |
| **Laricio Pine Sologne** | 0.11 | 200 | 0.11 | 150 | 0.10 | 150 |  |  |
| **Black Austrian Pine - South-East** | 0.11 | 150 | 0.12 | 100 | 0.09 | 100 | 0.08 | 100 |
| **Norway spruce - North East** | 0.12 | 250 | 0.10 | 250 | 0.11 | 200 | 0.10 | 200 |
| **Norway spruce - West Massif Cnt** | 0.09 | 250 | 0.09 | 200 | 0.11 | 150 |  |  |
| **Douglas - N-E Massif Central** | 0.14 | 250 | 0.14 | 200 | 0.13 | 200 |  |  |
| **Douglas - W Massif Central** | 0.14 | 250 | 0.13 | 250 | 0.13 | 250 |  |  |
| **Maritime Pine - SW - Landes** | 0.18 | 100 | 0.14 | 100 | 0.12 | 100 | 0.10 | 100 |
| **Maritime Pine - S-W, dune** |  |  | 0.16 | 100 | 0.12 | 100 | 0.11 | 100 |
| **Fir Jura** | 0.17 | 200 | 0.13 | 200 | 0.12 | 150 | 0.11 | 100 |
| **Spruce South of Massif Central** | 0.15 | 250 | 0.14 | 200 | 0.14 | 150 | 0.13 | 100 |
| **Fir North of the Alpes** | 0.10 | 450 | 0.10 | 400 | 0.10 | 250 | 0.09 | 150 |
| **Spruces North of the Alpes** | 0.07 | 650 | 0.09 | 350 | 0.08 | 300 | 0.09 | 200 |
| **Beech N-E** | 0.07 | 450 | 0.07 | 350 | 0.08 | 200 | 0.08 | 150 |
| **Beech N-W** | 0.07 | 400 | 0.07 | 350 | 0.08 | 250 | 0.09 | 150 |
| **Oak Center and West** |  |  |  |  | 0.09 | 200 |  |  |

At the regional scale, we assumed that trees growth can be simulated by using the three to four most representative species for coniferous and deciduous trees respectively. We used the inventories data provided by the IGN at the NUTS 3 level to select the 3 to 4 dominant species of each regional unit considered here, which represented more than 80% of all tree species in most regions. We assumed that this dominance pattern of deciduous and coniferous assemblages respectively has not significantly changed since the mid-19^th^ century. This assumption could be checked from figures on dominant tree species reported in 1908 and 1878. The regional r and K coefficients were then calculated as the weighted average of these most representative trees for coniferous and deciduous respectively. The same procedure was applied at the national level, pooling the different species reported for 1985. As statistical yearbooks do not provide information regarding the distribution of productivity classes of each tree among the total forest area, we fitted for each region the best productivity classes distribution for calculating standing biomass to empirical inventories data reported by the IGN for 1985, 1998 and 2011 (before 1985, no data on standing biomass were available at the regional level). These calculations were made using Microsoft Excel and associated VBA macros.

However, the r and K parameters derived from production table correspond to the pedoclimatic conditions in the 1960’s, when most of these tables were established. Dendrological measurements and other evidences clearly show that a significant increase in tree growth rate occurred since the beginning of the 20^th^ century in many regions of the North hemisphere (Boisvenue and Running, 2006), including France (Becker, 1989; Becker et al., 1994a, b; Badeau et al., 1996; Rathgeber et al., 1999; Charru et al., 2017). This increase has been attributed to climate change (Girardin et al., 2011), CO_2_ fertilization (Hickler et al., 2008), increased N deposition (Butterbach-Bahl and Gundersen, 2011), genomic selection (Resende et al., 2012) or the combination of all these factors (Oren et al., 2001). In order to account for this observed long term increase of forest NPP since the last century, we assumed that the r and K parameters may have linearly increased from the mid-19^th^ century to the estimated values of the 1960’s and then linearly increased again until now. We considered that possible r and K parameter values were in a range between 50% to 100% and 100% to 150% of their estimated value in the 1960’s, in 1850 and 2010 respectively. All possible combinations were tested at the national level by step of 5% and fitted to empirical inventories data of both coniferous and deciduous biomass reported by the IGN for 1985, 1998 and 2011. Optimization of parameters evolution lead to increasing r and K by 15% for coniferous from 1850 to 1960 then by 15% and 35% respectively for deciduous and coniferous from 1960 to 2015. In order to isolate the influence of changing environmental factor on biomass accretion, the time-dependency of r and K parameters was also tested through a sensitivity analysis with respect to a scenario with constant growth parameters (see section 2.8 below).

**2.3. Dynamics of standing biomass**

The dynamics of standing biomass (B) of a production forest at the regional scale depends on the harvest rate H (ton/ha/yr). This dynamics is described by the differential equation:

dB/dt = r.B.(1- B/K) – σ k_i_.ε_i_.B – H (2)

where ε_i_.and k_i_ represents respectively the proportion to total biomass and turnover time of the different organs, as listed in Table 1.

The standing biomass outputs by harvest are provided by region, separately for deciduous (hard wood) and conifers (soft wood), by forestry statistics. For France, long term chronicles were reconstructed from the archives of the *Bibliothèque historique du Ministère de l’Agriculture* (<http://www.unicaen.fr/mrsh/bibagri2/statistiques>) for the years 1862, 1878, 1882, 1892, 1908 and 1929, and from the statistical year books provided by AGRESTE and the IGN from 1947 to 2015. Figures provided by statistics refer only to exported wood (stems and branches). Corresponding losses of non-exploited organs (leaves, small branches, large and fine roots) left over on the forest floor are also considered, using the proportions reported in table 1a.

As the model is run on yearly steps, we interpolated the surface and harvest between the two closest documented dates for each region. Table 3 summarizes the sources and years of data collection. The initial standing biomass was initialized assuming that standing biomass was at an equilibrium in 1850, i.e., that NPP compensated losses through natural mortality and anthropogenic extraction. This assumption implies that the harvest rates reported in 1862 are reasonably representative for the preceding centuries.

**Table 3** *Summary of the sources and years of data collection. Green ticks mean that data were available at the regional level. Blue ticks mean that data were available at the national level, in that case data were interpolated at the regional level by linearly interpolating the regional distribution of the two closest date and then applying these regional distributions to the national figures. Purple ticks mean that the distinction between coniferous and deciduous was not available at the regional level but were calculated assuming a constant proportion to total surface or wood extraction with the closest data.*

| **Years** | **Total forest area** | **Decid forest area** | **Conif forest area** | **Total wood harvest** | **Decid wood harvest** | **Conif wood harvest** | **Decid standing biomass** | **Conif standing biomass** | **Data sources** |
| --- | --- | --- | --- | --- | --- | --- | --- | --- | --- |
| 1862 |  |  |  |  |  |  |  |  | Archives, Caen, France |
| 1878 |  |  |  |  |  |  |  |  | Archives, Caen, France |
| 1882 |  |  |  |  |  |  |  |  | Archives, Caen, France |
| 1892 |  |  |  |  |  |  |  |  | Archives, Caen, France |
| 1908 |  |  |  |  |  |  |  |  | Archives, Caen, France |
| 1910 |  |  |  |  |  |  |  |  | Archives, Caen, France |
| 1915 |  |  |  |  |  |  |  |  | Archives, Caen, France |
| 1920 |  |  |  |  |  |  |  |  | Archives, Caen, France |
| 1925 |  |  |  |  |  |  |  |  | Archives, Caen, France |
| 1929 |  |  |  |  |  |  |  |  | Archives, Caen, France |
| 1935 |  |  |  |  |  |  |  |  | Archives, Caen, France |
| 1940 |  |  |  |  |  |  |  |  | Archives, Caen, France |
| 1945 |  |  |  |  |  |  |  |  | Archives, Caen, France |
| 1947-2005 |  |  |  |  |  |  |  |  | Agreste, French Agricultural Ministry |
| 1950 |  |  |  |  |  |  |  |  | Archives, Caen, France |
| 1955 |  |  |  |  |  |  |  |  | Archives, Caen, France |
| 1960 |  |  |  |  |  |  |  |  | Archives, Caen, France |
| 1985 |  |  |  |  |  |  |  |  | IGN |
| 1998 |  |  |  |  |  |  |  |  | IGN |
| 2005-2015 |  |  |  |  |  |  |  |  | Agreste, French Agricultural Ministry |
| 2011 |  |  |  |  |  |  |  |  | IGN |

Note that two regions (*Grand Jura* and *Cantal Corrèze*) presented very high rates of wood extraction at some time periods, leading the model to predict biomass collapse even with the parameters for the highest productivity classes of dominant deciduous and coniferous species. Consequently, for these two regions the growth rate *r* and carrying capacity *K* were artificially increased by 3 to 9%.

1. **The Litter and Soil Organic Matter module**

**3.1. Litter structure and dynamics**

Following the FORCLIM-D model as described by Perruchoud et al., (1999) and Liski et al., (2002), four compartments of litter were considered: coarse woody litter, large roots and branches litter, fine root litter and foliage litter (Fig. 4). These compartments were fed by natural losses of the different tree organs, as well as by material left over on the forest floor after felling or cutting. Each of these litter compartments is decaying at a specific rate (see Table 4).

**Figure 4**. *Functional relationships between the different organs in standing biomass and the litter compartments. Black arrows represent natural loss processes; red arrows represent additional losses related to felling and cutting, during which events stem and most branches are exported, while corresponding roots and foliage are left over on the forest floor.*

Natural losses of standing biomass to litter are calculated using the proportion of each organ in total biomass and their turnover times mentioned in Table 1.

Losses to litter by felling and cutting correspond to the parts of trees that are not exported with harvest but left on the forest floor. These are calculated as a fraction of harvested wood, as indicated in table 5.

**Table 4**. *Decay rate (mineralization to CO_2_ and transfer to fast humus) of the different litter compartments. (data from Liski et al 2002).*

|  |  | **deciduous** | **conifers** |
| --- | --- | --- | --- |
| coarse woody litter | yr^-1^ | 0.12 | 0.028 |
| branches & large roots | yr^-1^ | 0.22 | 0.22 |
| foliage & fine roots | yr^-1^ | 0.35 | 0.25 |

**Table 5** *Losses at felling and cutting of different organs in proportion of exported wood (calculated from the proportion of each organ in total tree biomass (Table 1), except for branches for which a ratio non-harvested to harvested of 20% is assumed)*

|  |  | **deciduous** | **conifers** |
| --- | --- | --- | --- |
| stem | Fraction of exported harvest | 0 | 0 |
| branches | Fraction of exported harvest | 0.05 | 0.07 |
| large roots | Fraction of exported harvest | 0.1 | 0.09 |
| fine roots | Fraction of exported harvest | 0.02 | 0.015 |
| foliage | Fraction of exported harvest | 0.02 | 0.07 |
| total |  | 0.19 | 0.24 |

The decay rate of foliage and fine roots litter, the most reactive compartments of litter, is dependent on temperature and moisture: Liski et al. (2002) have fitted the following relationship to the data collected by Berg et al. (1993) with annual mean temperature and the difference between precipitation and potential evapotranspiration (PET) from May to September as explanatory variables:

$k_{foliage \& fine roots litter}=0.35\times resp\times0.25\times\left( 1+0.094\times\left[ T-4 \right]+0.0023\times\left[ M+50 \right] \right)$ (3)

with T = annual average temperature (°C)

M = difference between precipitation and PET from May to September (mm)

(see below for the long term and regional variations of T and M)

In historical periods and in some regions, a significant fraction of litter (mostly foliage, small branches and brigs) was subject to harvest or grazing, as the forest was closely associated with the agricultural system. A rate of harvest up to 50% of the foliage litter production until World War II was considered by Perruchoud (1999) based on data from and Bürgi (1998). Here we applied these same factors to all French regions from 1850 to 1940 and then assume a gradual reduction of litter harvest and grazing until the end of the 1970’s down to 0%.

**3.2. Humus dynamics**

Two compartments of humus are considered (Fig. 1 and 2), differing by their turnover rate.

The decay of litter results for the largest part in CO_2_ emission, while a fraction is converted into a fast humus compartment. This latter fraction is listed in Table 6.

**Table 6**. *Fraction of* *litter* *decay converted into fast humus (data from Liski et al 2002).*

|  |  | **Deciduous and conifers** |
| --- | --- | --- |
| coarse woody litter | fraction | 0.45 |
| branches & large roots | fraction | 0.45 |
| fine roots | fraction | 0.27 |
| foliage | fraction | 0.51 |

Fast humus decay rate (a, yr^-1^) is dependent on mean annual temperature (T) as a sole climatic control according to the following relationship (Trumbore et al., 1996):

Turnover time = 1/a = 138. exp (-0.11 x T) (4)

The slow humus compartment is characterized by a decay rate equal to 0.007 times the decay rate of the fast humus compartment (Perruchoud et al., 1999; Liski et al., 2002). This slow humus is fed by a fraction 0.0033 of fast humus decay. Given the value of these parameters, the steady state ratio of slow to fast humus is expected to be 0.007/0.0033 = 2.1. The initial stock of litter, and humus was calculated at the equilibrium in 1850, i.e., assuming that C inputs from biomass to soil and litter compensated losses through mineralization and transfer to other pools.

**4. Land use changes**

The land-use change from agricultural land (cropland and grassland) to forest area that may have occurred from 1850 to 2015 was accounted for if, for a given year, agricultural land lost surfaces while forest land gained surfaces, considering the conversion from one to the other as the smallest absolute surface change. The fast and slow humus content in agricultural soils in each French region from 1850 to 2015 is known from the previous work by Le Noë et al., (2019 a, b) coupling the AMG (Saffih-Hdaddi and Mary, 2008; Clivot et al., 2019) and the GRAFS approach (Le Noë et al., 2018). Biomass and litter content of agricultural soil are neglected. For each time step from year n to n+1, the fraction ɛ of forest area which was converted from agricultural land is calculated, and the forest per area stock of each compartment (C_F_) is corrected according to the formula:

C_Fn+1_ = (C_Fn_ +ΔC_Fn_)

$C_{F n+1}= \left( {C_{F n}+\Delta C}_{F n} \right)\times\frac{1}{1+ɛ}+ \left( C_{A n} \right)\times\frac{ɛ}{1+ ɛ}$ (5)

Where C_A n_ is the corresponding per area stock of carbon (biomass, litter, fast and slow humus) in agricultural land at year n. The values of ε are generally close to zero, and reach a few percent only during limited periods in specific regions. Considering land use changes thus introduces only a second-order correction during these periods. Note that if a land-use change occurs and impact forest SOC, this will be translated on a per hectare basis as a *dilution* of SOC stock due to the inputs of SOC from grassland and cropland which generally have lower SOC content.

**5. Climate Variable**

As stated above, mineralization rate of fast and slow humus are depending on mean annual temperature. Foliage and small root litter also depends on the difference between rainfall and potential evapotranspiration between May and September, representing the summer excess or deficit of pluviometry. These two forcing climate variables were calculated for the different regions of France over the period 19909-2010 from the MESAN data base, a European high-resolution surface reanalysis ([Häggmark et al. 2000](https://journals.ametsoc.org/doi/10.1175/JHM-D-17-0018.1); [Landelius et al. 2016](https://journals.ametsoc.org/doi/10.1175/JHM-D-17-0018.1)). ETP has been calculated using the formula proposed by Oudin et al (2005):

PET(T) = Re/(l.r.100) * (T + 5)

with PET in mm/day

T in °C

Re(t)= extraterrestrial radiation (MJ/m2/day)

l latent heat of vaporization (MJ/kg)

r water density (1000 kg/ m3)

**Figure 5.** *Mean annual temperature in 2010 and summer excess or deficit pluviometry in the different regions of France clacualted from the data of the MESAN data base (Landelius et al 2016).*

To calculate these forcing climate variables for the historical period, the approach developed by Le Noë et al (2019) has been applied, based on the available data of temperature anomaly with respect to the period 1990-2010. The above formula is used to calculate PET in function of this temperature anomaly. In the absence of evidence for significant variation, interannual average pluviometry is considered invariant over the whole period, as assumed by Garnier et al (2019):

**Figure 6.** **a.** *Temperature anomaly with respect to the average of 1990-2010 period since 1850.*

**b.** *Summer excess (or deficit) pluviometry over ETP over the period 1850-2010 in different region of France, calculated from the data of the MESAN data base and the temperature anomaly.*

**6. Sensitivity of the model to key assumptions**

We performed sensitivity analyses to assess the significance of three key model assumptions (see figure in SOM1). (i) The hypothesis made about litter extraction through harvest and grazing in historical periods was tested by assuming, besides the standard hypothesis of 50% of annual litter production until 1940, two scenarios with no litter extraction and 100% litter extraction, respectively. The results showed SOC values 75% higher and 75% lower with respect to the standard hypothesis in 1850. However, this relative gap was much lower in 2015, reaching SOC stocks merely 23% lower and higher in the scenario with no litter grazing and 100% of litter grazing respectively. Our standard hypothesis provided SOC estimations closer to the measured values than did the two scenarios, thus suggesting that this hypothesis is more accurate than the other two. However, this sensitivity assessment points towards the relevance of forest uses for SOC stocks in our approach.

(ii) The sensitivity of the model towards productivity class distribution was also explored with productivity classes distributed according to the ratios 1:1:1:1 and 1:2:2:1, instead of calibrating this distribution. Both scenarios led to higher estimations of C stocks in biomass and SOC (from 34 % to 55 %), both in 1850 and 2015. This simulation indicates that optimization of productivity classes distribution is key for simulating reliable outputs.

(iii) The explorations of lower and higher initial standing biomass influenced C stocks both in biomass and SOC with respect to the standard hypothesis of steady state standing biomass in 1850. However, the relative gap diminished significantly over time and reached only 1 to 3% in 2015, thus suggesting that the standard hypothesis of steady state in 1850 is reasonable.


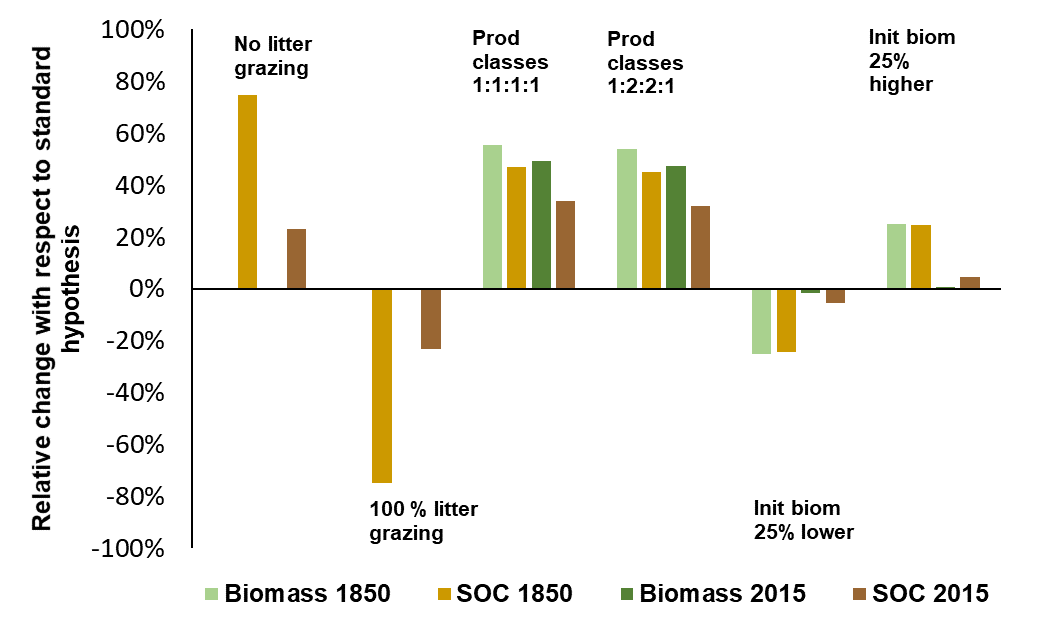


Figure 7. *Sensitivity analysis of the main model hypotheses. Influences of major model assumptions on biomass and SOC stocks estimations are tested at the level of France in 1850 and 2015.*

**7. Calibration and validation of the model**

CRAFT modelled results of C stocks in standing biomass were tested against empirically observed data (IGN, 2018) for coniferous and deciduous trees in 1985, 1998 and 2011 at the regional scale. As expected, the model revealed good performance since calculated coniferous and deciduous standing biomass were significantly correlated (p <0.05) with the observed data, in terms of total stocks (MtonC), biomass density (tonC ha^-1^) and variation of total stocks (∆MtonC 1985-2011) (see SOM1 for additional figures). Only in two Mediterranean regions (*Côte d’Azur* and *Grand Marseille*) was the model unable to consistently mimic the C dynamics in deciduous biomass, suggesting that the production tables may not be suited for deciduous trees in Mediterranean regions. These regions were removed from the correlation graph in figure 7.c.


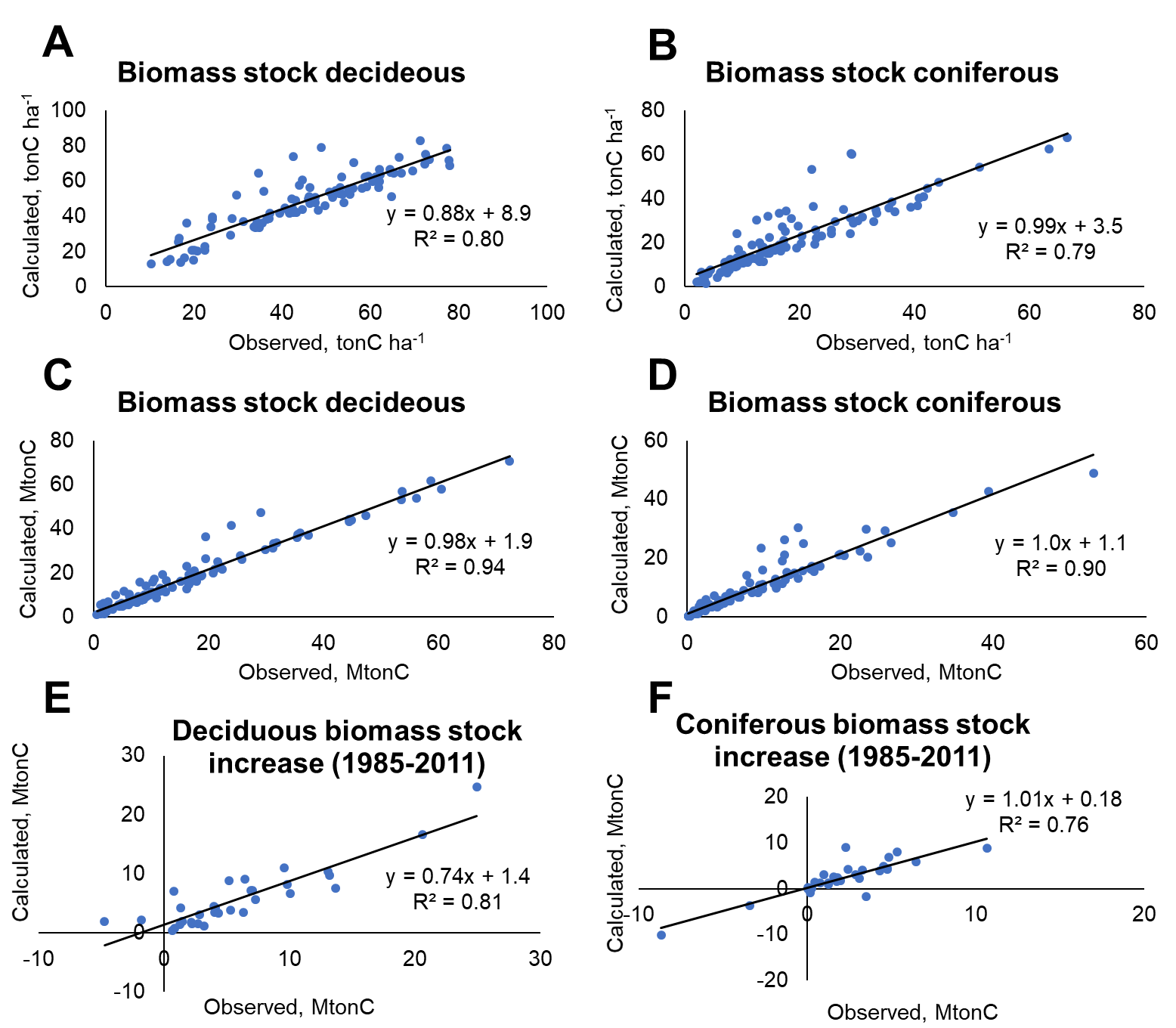


**Figure 8.** *Validation of the model against observed data from the IGN inventories in 1985, 1998 and 2011. Panel A-B, C-D and E-F respectively provides validation of the simulated data against observed data in terms of MtonC, tonC ha^-1^ yr^-1^ and change in biomass C stock (Δ MtonC). Linear regressions are all significant (P < 0,05).*

The implementation of the model at the regional level enabled us to compare the national trends of forest C stocks when considering France as the aggregation of all the 33 territories, and France as a single large region itself, using average data on temperature, drought, wood extraction, forest area, average distribution of dominant coniferous and deciduous species and coefficients for productivity classes distribution. Outputs from both calculation approaches revealed very similar results, thus indicating no or very little scale effect of the model (Fig. 8).


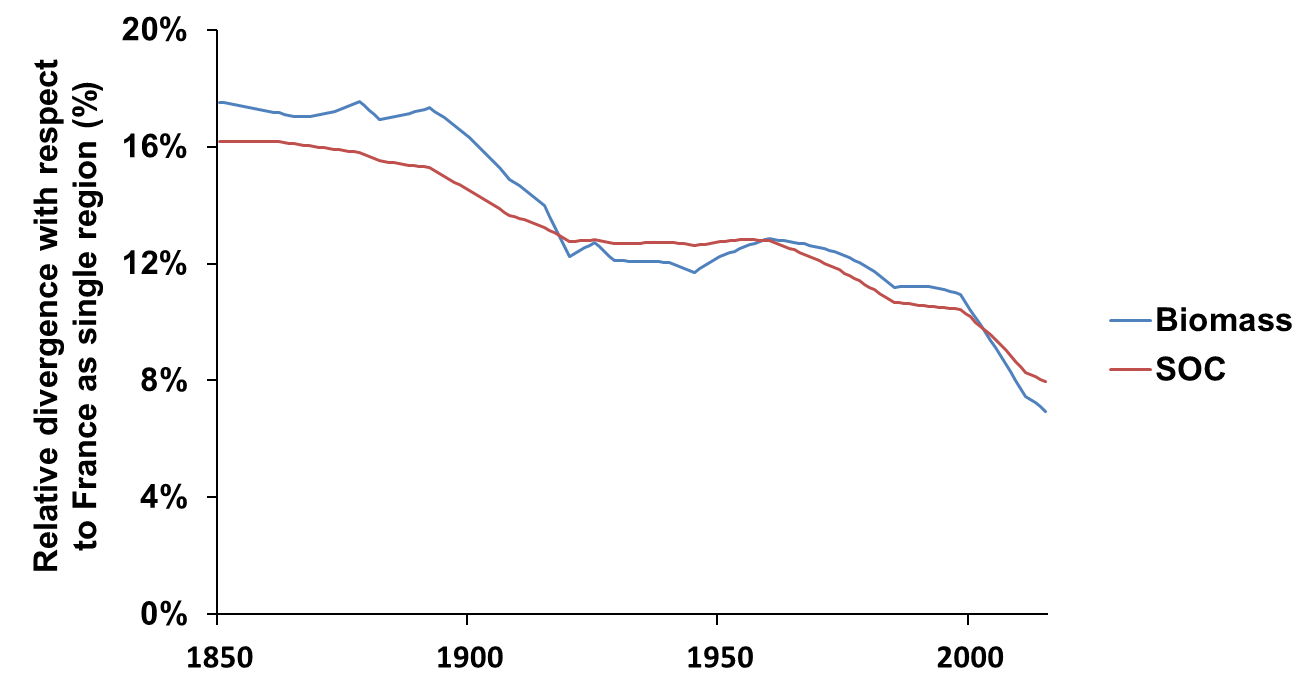


**Figure 9.** *Relative divergence of the biomass and SOC stocks in France simulated as the aggregation of all regions with respect to the simulation of France as a single region.*

**8. Summary: a short instruction list for future users**

The CRAFT model is designed for quantifying C reservoir accretion in forest transition. Therefore, before using the model, user should ensure that they will have inputs data over the period which covers forest transition. As shown in the main paper, the model is suited for regional and national studies in temperate, mountainous, continental and Mediterranean areas. The inputs data required are:

- Forest area, distinguishing as often as possible deciduous and coniferous areas
- Wood harvest, distinguishing as often as possible deciduous and coniferous wood harvest
- Chronicles of Temperature
- Records of precipitation
- SOC contents in agricultural land (chronicles if available and if not recent data)

The parametrization data required are:

- Yield production tables specific to the area under study
- Recent estimations of standing coniferous and deciduous biomass.

Collecting all these data represents the **first step** to run the model. Next steps are:

**2^nd^ Step**: derive logistic function from yield tables for the most commonly encountered species. The excel files provided in SOM2 can be used for that step.

**3^rd^ Step**: Collect and organize inputs data in an excel sheet. The excel files provided in SOM3 can be used for that step.

**4^th^ Step**: Enter the parameters of the logistic function calculated in step 2 for the most common coniferous and deciduous trees of the area under study. At least 4 distribution classes should be calculated. The excel files provided in SOM3 can be used for that step.

**5^th^ Step**: Optimize the productivity classes distribution and time-evolution of r and K parameters by running a routine using Macro excels so that simulated data best fit to the available observed data. The excel files provided in SOM3 can be used for that step.

**6^th^ Step**: Run the model and analyze the data as you wish.

**References**

Badeau V, Becker M, Bert D et al. (1996) Long-term growth trends of trees: ten years of dendrochronological studies in France. In: Growth Trends in European Forests (eds Spiecker H, Mielikainen K, Köhl K, Skovsgaard JP), pp. 167–181. Springer, Berlin.

Becker M. (1989). The role of climate on present and past vitality of silver fir forest in the Vosges mountains of NE France. Can J. Forest Res 19 (1989) 1110-1117

Becker M., Bert G.D.,Bouchon J., Picard J.F.,Ulrich E. (1994).Tendances à long terme observées dans la croissance de divers feuillus et résineux du NE de la France depuis le milieu du 19e s. Rev.For. Fr. XLVI 335-341.

Becker M., Niemen T.M., Gérémia F., (1994). Short-term variations and long term changes in oak productivity in NE France. The role of climate and atmospheric CO2. Ann. Sci.For. 51: 477-492

Berg, B., Berg, M.P., Bottner, P., Box, E., Breymeyer, A., Calvo De Anta, R., Couteaux, M., Escudero, A., Callardo, A., Kratz, W., Madeira, M., Malkonen, E., McClaughery, C., Meentemeyer, V., Munoz, F., Piussi, P., Remecle, J., Virzo De Santo, A., 1993. Litter mass loss rates in pine forests of Europe and Eastern United States: some relationships with climate and litter quality. Biogeochemistry 20, 127–159.

Billen, G., Lassaletta, L., Garnier, J., (2014). A biogeochemical view of the global agro-food system: Nitrogen flows associated with protein production, consumption and trade. *Global Food Security* 209–219. DOI: [10.1016/j.gfs.2014.08.003](https://www.sciencedirect.com/science/article/abs/pii/S2211912414000315).

Boisvenue C, Running SW (2006) Impacts of climate change on natural forest productivity - evidence since the middle of the 20th century. Glob Chang Biol 12:862–882

Bürgi M (1998). A case study of forest change in the Swiss lowlands. Landscape Ecology 14: 567–575

Butterbach-Bahl K & Gundersen P (2011). Nitrogen processes in terrestrial Ecosystems. In The European Nitrogen Assessment, Sutton M, Howard C, Erisman JW, Billen G, Bleeker A, Grennfelt P, van Grinsven H, Grizzetti B. Chapter 6, pp 99-125. Cambridge University Press.

Charru M, Seynave I, Hervé JC, Bertrand R, Bontemps JD (2017). Recent growth changes in Western European forests are driven by climate warming and structured across tree species climatic habitats. Annals of Forest Science 74: 33. DOI 10.1007/s13595-017-0626-1

Clivot, H., Mouny, J.-C., Duparque, A., Dinh, J.-L., Denoroy, P., Houot, S., Vertès, F., Trochard, R., Bouthier, A., Sagot, S., Mary, B., (2019). Modeling soil organic carbon evolution in long-term arable experiments with AMG model, Environmental Modelling & Software, 118, 99-113. DOI: 10.1016/j.envsoft.2019.04.004.

Dupouey et al., (2000) Stocks et Flux de carbone dans les forêts françaises. Revue Forestière Française – numéro spécial 2000.

Duvigneaud P. (1971). Concepts sur la productivité primaire des écosystèmes forestiers . In Unesco, Productivité des écosystèmes forestiers. Actes Coll. Bruxelles. Ecologie et Conservation, 4.

Eggleston, H.S., Intergovernmental Panel on Climate Change, National Greenhouse Gas Inventories Programme, and Chikyū Kankyō Senryaku Kenkyū Kikan (2006). 2006 IPCC guidelines for national greenhouse gas inventories.

Garnier, J., Le Noë, J., Marescaux, A., Sanz-Cobena, A., Thieu, V., Silvestre, M., Billen, G., (2019). Long-term changes in greenhouse gas emissions from French agriculture (1852-2014): from traditional agriculture to conventional intensive systems. *Sci. Total Environ.* 660, 1486-1501. DOI: [10.1016/j.scitotenv.2019.01.048](https://www.ncbi.nlm.nih.gov/pubmed/30743941)

Girardin MP, Bernier, PY, Raulier F, Tardif JC, Conciatori F, Guo XJ (2011). Testing for a CO2 fertilization effect on growth of Canadian boreal forest. J. Geophy Res. 116, GO1012. Doi:10.1029/2010JG001287

Häggmark, L., K.-I. Ivarsson, S. Gollvik, and P.-O. Olofsson, 2000: MESAN: An operational mesoscale analysis system. *Tellus*, **52A**, 2–20, doi:[https://doi.org/10.3402/tellusa.v52i1.12250](https://doi.org/10.3402%2Ftellusa.v52i1.12250).

Hickler T, Smith B, Prentice C, Mjöfors K, Miller P, Arneth A, Sykes MT (2008). CO2 fertilization in temperate FACE experiments not representative of boreal and tropical forests. Global Change Biology. 14, 1531–1542, doi: 10.1111/j.1365-2486.2008.01598.x

Kicklighter, D.W. et al. (1999). A first-order analysis of the potential role of CO2 fertilization to affect global carbon budget: a comparison of four terrestrial biosphere models. Tellus B 51, 343-366 (1999).

Landelius, T., P. Dahlgren, S. Gollvik, A. Jansson, and E. Olsson, 2016: A high-resolution regional reanalysis for Europe. Part 2: 2D analysis of surface temperature, precipitation and wind. *Quart. J. Roy. Meteor. Soc.*, **142**, 2132–2142, doi:[https://doi.org/10.1002/qj.2813](https://doi.org/10.1002%2Fqj.2813).

Le Noë, J., Billen, G., Mary, B., Garnier, J., (2019b). Drivers of long-term carbon dynamics in cropland: a bio-political history (France, 1852-2014). *Environ. Sci. & Pol*. 93, 53-65. [DOI : 10.1016/j.envsci.2018.12.027](https://doi.org/10.1016/j.envsci.2018.12.027)

Liski, J., Perruchoud, D., Karjalainen, T. (2002). Increasing carbon stocks in the forest soils of western Europe. Forest Ecology and Management, 169, 159-175. <https://doi.org/10.1016/S0378-1127(02)00306-7>

Nabuurs, G.-J., Schelhaas, M.-J., Mohren, G., Frits M.J., Field, C.B. (2003). Temporal evolution of the European forest sector carbon sink from 1950 to 1999. *Glob. Change Biol*. *9*, 152–160.

<https://doi.org/10.1046/j.1365-2486.2003.00570.x>

Oren R, Ellsworth DS, Johnsen KH, Phillips N, Ewers BE, Maier C, Schaefer KVR, Heather McCarthy H, Hendrey G, McNulty SG & Katul GG.(2001). Soil fertility limits carbon sequestration by forest ecosystems in a CO2-enriched atmosphere. Nature. 411. 469-472.

Oudin L, Hervieu F, Michel C, Perrin C, Andréassian V, Anctil F, Loumagne C. (2005). Which potential evapotranspiration input for a lumped rainfall-runoff model?. Part 2. Towards a simple and efficient potential evapotraspiration model for rainfall-runoff modelling. Journal of Hydrology 303: 290-306.

Perruchoud, D., Joos, F., Fischlin, A., Hajdas, I., Bonani, G., 1999. Evaluating timescales of carbon turnover in temperate forest soils with radiocarbon data. Global Biogeochem. Cycles 13, 555–573.

Rathgeber, C., Guiot, J., Roche, P., Tessier, L., (1999). Augmentation de productivité du chêne pubescent en région méditerranéenne française. Ann. For. Sci. 56, 211–219

Rathgeber C, Nicault A, Kaplan JO, Guiot J. (2003). Using a biogeochemistry model in simulating forests productivity responses to climatic change and [CO2] increase: example of *Pinus halepensis* in Provence (south-east France). Ecological Modelling 116: 239-255

Saffih-Hdadi K. and Mary B. (2008). Modeling consequences of straw residues export on soil organic carbon. *Soil Biology & Biochemistry*, 40, 594-607

Saint-André et al. (2010)., Chapter 4: Estimating carbon stocks in forest stands: Chapter 4 Estimating carbon stocks in forest stands: 1. Methodological developments. In Lousteau Denis (Ed.): Forests, carbon cycle and climate change, Versailles: Quae, 2010 311pp.

Trumbore SE, Chadwick OA, Amundson R. 1996. Rapid exchange between soil carbon and atmospheric carbon dioxide driven by temperature change. Science 272:393–6.

Vannière, B., (1984). Tables de production pour les forêts français*es*. École Nationale du Génie Rural, des Eaux et des Forets, Nancy, 2^ème^ édition. ISBN – 2 – 85710 – 016 – 7.
